# Obesity as the Upstream Driver of Inflammation and Insulin Resistance in Hyperuricaemia

**DOI:** 10.1002/edm2.70224

**Published:** 2026-04-17

**Authors:** Mohammad Riashad Monjur

**Affiliations:** ^1^ Department of General Medicine Campbelltown Hospital, South Western Sydney Local Health District Campbelltown New South Wales Australia

Dear Editor,

Huang et al. [1] reported that systemic inflammation (modified inflammation [mINFLA] score) and insulin resistance (triglyceride‐glucose [TyG] index) are independently and jointly associated with hyperuricaemia (HUA) risk, with the TyG index mediating approximately 15% of the inflammation‐HUA relationship. The authors provide a thorough analysis of these interacting pathways. This correspondence proposes that for a substantial subset of individuals with overweight or obesity, adiposity may function not as another concurrent pathway but as the unifying upstream driver of both inflammation and insulin resistance in HUA.

The authors' own supplementary mediation analysis supports this interpretation: body mass index (BMI) mediated 44.2% of the inflammation‐HUA association—nearly three times the proportion mediated by the TyG index (15.4%). This striking disparity suggests that in individuals with excess adiposity, obesity sits hierarchically above both inflammation and insulin resistance, generating each as a downstream consequence of adipose tissue dysfunction. Visceral adiposity drives pro‐inflammatory cytokine secretion (tumour necrosis factor‐α [TNF‐α], interleukin‐6 [IL‐6]) [2] while simultaneously impairing insulin signalling through reduced adiponectin and ectopic lipid deposition [3]. In this subset, adjusting for BMI as a covariate, rather than positioning it as the upstream variable, may obscure its central role and risk distorting mediation estimates .

This reframing carries therapeutic implications. For individuals with overweight or obesity and HUA, targeting weight reduction through lifestyle modification and pharmacotherapy, including incretin‐based therapies, may simultaneously ameliorate inflammation, insulin resistance and uric acid dysregulation [4, 5], addressing the root cause rather than individual downstream pathways.

## Author Contributions

**Mohammad Riashad Monjur:** conceptualization, writing – original draft, writing – review and editing.

## Conflicts of Interest

The author declares no conflicts of interest.

## Data Availability

Data sharing not applicable to this article as no datasets were generated or analysed during the current study.
